# Habitat connectivity and resource selection in an expanding bobcat (*Lynx rufus*) population

**DOI:** 10.7717/peerj.12460

**Published:** 2021-11-11

**Authors:** Viorel D. Popescu, Madeline Kenyon, Ryan K. Brown, Marissa A. Dyck, Suzanne Prange, William E. Peterman, Catherine Dennison

**Affiliations:** 1 Biological Sciences, Ohio University, Athens, OH, United States; 2 Center for Environmental Research, University of Bucharest, Bucharest, Romania; 3 Appalachian Wildlife Research Institute, Athens, OH, United States of America; 4 School of Environment and Natural Resources, Ohio State University, Columbus, OH, United States; 5 Division of Wildlife, Ohio Department of Natural Resources, Columbus, OH, United States of America

**Keywords:** Resource selection function, Circuitscape, Home range, Spatial scale, Habitat suitability, Dispersal

## Abstract

Terrestrial carnivores are among the most imperiled species worldwide, yet some species are resilient and are recovering in human-dominated landscapes after decades or centuries of absence. Bobcat (*Lynx rufus*) populations were extirpated from much of Midwestern US in the mid-1800’s, and are currently expanding and recolonizing their former range. In this study, we investigated multi-scale habitat selection for Ohio’s expanding bobcat population, and examined habitat connectivity in order to evaluate the conduits for dispersal statewide. We used citizen observations collected between 1978 and 2019 and logistic regression to evaluate population-level habitat selection, and GPS telemetry data for 20 individuals collected between 2012 and 2014 and a distribution-weighted exponential Resource Selection Function to evaluate individual-level habitat selection within home ranges. At the population level, bobcats selected for higher amounts of forest and pasture (at a 50 km^2^ scale) and herbaceous vegetation (at 15–50 50 km^2^ scales), thus overall heterogeneous forested habitat. At individual (home range) level, bobcats selected for forested habitats with low road density and farther away from high traffic roads; they also showed weak selection for open habitat at the home range level. Male home ranges were significantly greater than female home ranges. Lastly, we used the population-level spatial outputs (*i.e*. habitat suitability map) to parameterize habitat connectivity models using circuit theory in the program Circuitscape. We tested three relationships between habitat suitability and resistance to movement and used a subset of data on potential dispersing individuals to evaluate which relationship performed best. All three relationships performed almost equally well, and we calculated a weighted averaged connectivity map as our final map. Habitat was highly permeable to movements between core areas of two genetically distinct subpopulations located in southeastern Ohio. We also identified potential dispersal corridors from the core areas to other regions of Ohio dominated by agriculture and suburban development *via* forested riparian corridors. Overall, our analysis offers new information on habitat selection and connectivity in a rebounding felid population and offers important ecological information for wildlife management strategies. We recommend that the suitability and connectivity models should be periodically updated until the population reaches an equilibrium, and be integrated with data from neighboring states for a comprehensive assessment of a conservation success story.

## Introduction

Terrestrial carnivores are some of the most imperiled species worldwide because of their large home range requirements, high metabolic demands, sensitivity to habitat degradation, and persecution by humans (Palomares et al., 1999; Ripple et al., 2014). However, some terrestrial carnivore species are resilient to human pressures and in recent decades have rebounded in many parts of the world. Although terrestrial predators often need intact landscapes devoid of human impacts to recover (Gilroy, Ordiz & Bischof, 2015), the recovery of predators in many landscapes has shown that some carnivores are capable of effectively recolonizing human-dominated landscapes and coexisting with humans. In North America, decades of management efforts to recover wildlife populations using adaptive management principles and the North American Model of Wildlife Conservation (Organ et al., 2012) have resulted in increases and expansions of many carnivore species (*e.g*. wolves in Western and Midwestern US, bobcats and fishers in Midwestern US).

Natural expansion and colonization of former or new ranges are dependent on both the capacity and suitability of the habitat to support resident populations and landscape connectivity to maintain gene flow and dispersal processes (Malaney et al., 2018). As such, conservation and management of carnivore populations requires both assessments of resource selection at multiple scales (Boyce, 2006; Johnson et al., 2006) and an understanding of how human impacts affect dispersal and demographic processes (*e.g*. sources of mortality, barriers to dispersal; (Crooks et al., 2011; Proctor et al., 2012; Litvaitis et al., 2015)). Due to their large space use requirements, high mobility, and territoriality, carnivore conservation is particularly challenging in areas with high road densities (Basille et al., 2013; Poessel et al., 2014; Litvaitis et al., 2015; Bencin et al., 2019). Both high-traffic roadways and conversion of natural habitat to other land cover types (agriculture, urban sprawl) may limit the ability of species to recolonize their former ranges and maintain population connectivity across broad geographic extents.

The US Midwest is undergoing a natural experiment in native and invasive carnivore population expansion. Coyotes (*Canis latrans*) have expanded their range into this region starting in the early 1900s (Hody & Kays, 2018). Following extirpation from large portions of the Midwest by the mid-1800s due to habitat destruction and overharvesting, bobcats (*Lynx rufus*) have been recolonizing portions of their former range in this region after a century of absence (Nielsen & Woolf, 2002a; Roberts & Crimmins, 2010; Prange & Rose, 2020). Broad-scale data collected by management agencies (*e.g*. roadkill animals) and citizen observations (*e.g*. camera trap and live sightings) suggest that bobcat populations are expanding spatially and increasing in abundance. However, their expansion may be limited by human (non-harvest) impacts, such as road mortality (Nielsen & Woolf, 2002a; Bencin et al., 2019). Additionally, habitat can become limiting as intensive, row-crop agriculture dominates this region of the United States. As such, understanding the limiting factors affecting the distribution and habitat use on expanding bobcat populations at multiple spatial scales can improve the development and implementation of management and conservation plans and decisions to ensure long-term population viability (Hurlbert & Jetz, 2007; Reed et al., 2017b).

We focused on Ohio’s recovering bobcat population, and implemented habitat selection analyses at two levels: statewide (population level) using citizen science data and individual (home range level) using GPS telemetry data. We then used the population level outputs to parameterize circuit theory-based connectivity models (McRae et al., 2008) and identify potential movement pathways between population clusters across the state (Anderson, Prange & Gibbs, 2015). In Ohio, bobcats were extirpated by the 1850s due to overharvesting and habitat conversion to agriculture, and recolonization occurred sequentially with two genetically distinct subpopulations (as of 2012) in southern and eastern Ohio; the eastern population was determined to be self-sustaining (with founder animals from West Virginia), whereas the southern population (with founder population from Kentucky), is thought to be dependent on immigration (Anderson, Prange & Gibbs, 2015). However, these two subpopulations are only ~200 km apart, with no physical barriers separating them, and recent camera trap and roadkill data (Bencin et al., 2019) depict a more even distribution across the broader southeastern Ohio region, suggesting that admixture may be occurring. Bobcats were listed as ‘endangered’ in Ohio until 2012, downlisted to ‘threatened’, then delisted in 2014, with no trapping or hunting allowed. There is interest in opening a regulated harvest season for bobcats in Ohio, therefore evaluating whether differences in resource selection across different subpopulations occur and whether the habitat is permeable to movement between subpopulations, has important implications for future delineation of areas that could sustain limited harvest. In addition, because the number of bobcat sightings in Ohio increased in the last decade, including in areas with little-to-no forest cover outside of southeastern Ohio, identifying conduits of dispersal across the state could further inform decision-making on harvest areas.

The overarching goal of this study was to gain insights into the resource selection and regional connectivity of an expanding bobcat population in order to inform conservation and management decision-making that ensures long-term population sustainability. Specifically, the objectives of this research were to: (1) evaluate population-level selection across a heterogeneous mixed-use US Midwestern landscape using citizen science observations collected by the Ohio Division of Wildlife, (2) evaluate individual-level selection at the home range scale using GPS telemetry data, and (3) determine conduits for movement in forested and agricultural landscapes using circuit-theory models and results from Objective 1. We hypothesized that at the population-level, bobcats will select for forested (Lovallo & Anderson, 1996; Tucker, Clark & Gosselink, 2008; Reding et al., 2013) and heterogeneous landscapes (forest interspersed with open herbaceous habitat, including strong selection for open herbaceous habitat during summer (Kamler & Gipson, 2000; Linde et al., 2012)) and areas farther from high-traffic roads (Bencin et al., 2019). At the home range level, we hypothesized that topography, proximity to high traffic roadways and habitat heterogeneity would influence habitat selection (Kolowski & Woolf, 2002; Prange & Rose, 2020). Based on previous bobcat movement and habitat use studies (Johnson, Walker & Hudson, 2010; Reding et al., 2013; Hughes et al., 2019), we expect natural areas along river corridors or other natural habitats to act as conduits for dispersal in heavily altered (agricultural) areas. Overall, this suite of resource selection and connectivity analyses can help elucidate aspects of bobcat spatial ecology and potential limiting factors to population expansion, as well as provide important information for developing and implementing population models to inform the impacts of potential harvest on population persistence (LaRue & Nielsen, 2016).

## Methods

### Overview

We implemented population-level habitat selection using a dataset of verified bobcat sightings (camera trap, personal observation, incidental trapping) collected by the Ohio Department of Natural Resources, Division of Wildlife (ODOW) between 2010 and 2019 (out of a dataset spanning 1960–2019) and logistic regression to evaluate macro-scale land cover predictors of occurrence and predict current Ohio-wide habitat suitability. More than 95% of the observations in the original dataset were collected after 2000, and 91% were recorded after 2010. We implemented individual-level (within home range) habitat selection analyses using a GPS telemetry dataset collected between 2012 and 2014 in two subpopulations (Prange & Rose, 2020), and a weighted-distribution resource selection approach (Lele & Keim, 2006; Lele, 2009) using fine-scale predictors, such as distance to roads and streams, road density and habitat types. Lastly, we used the habitat suitability map developed based on the population-level selection analysis as the resistance (or permeability) layer to parameterize a state-wide habitat connectivity model.

### Long-term bobcat sighting data

**Figure 1:**
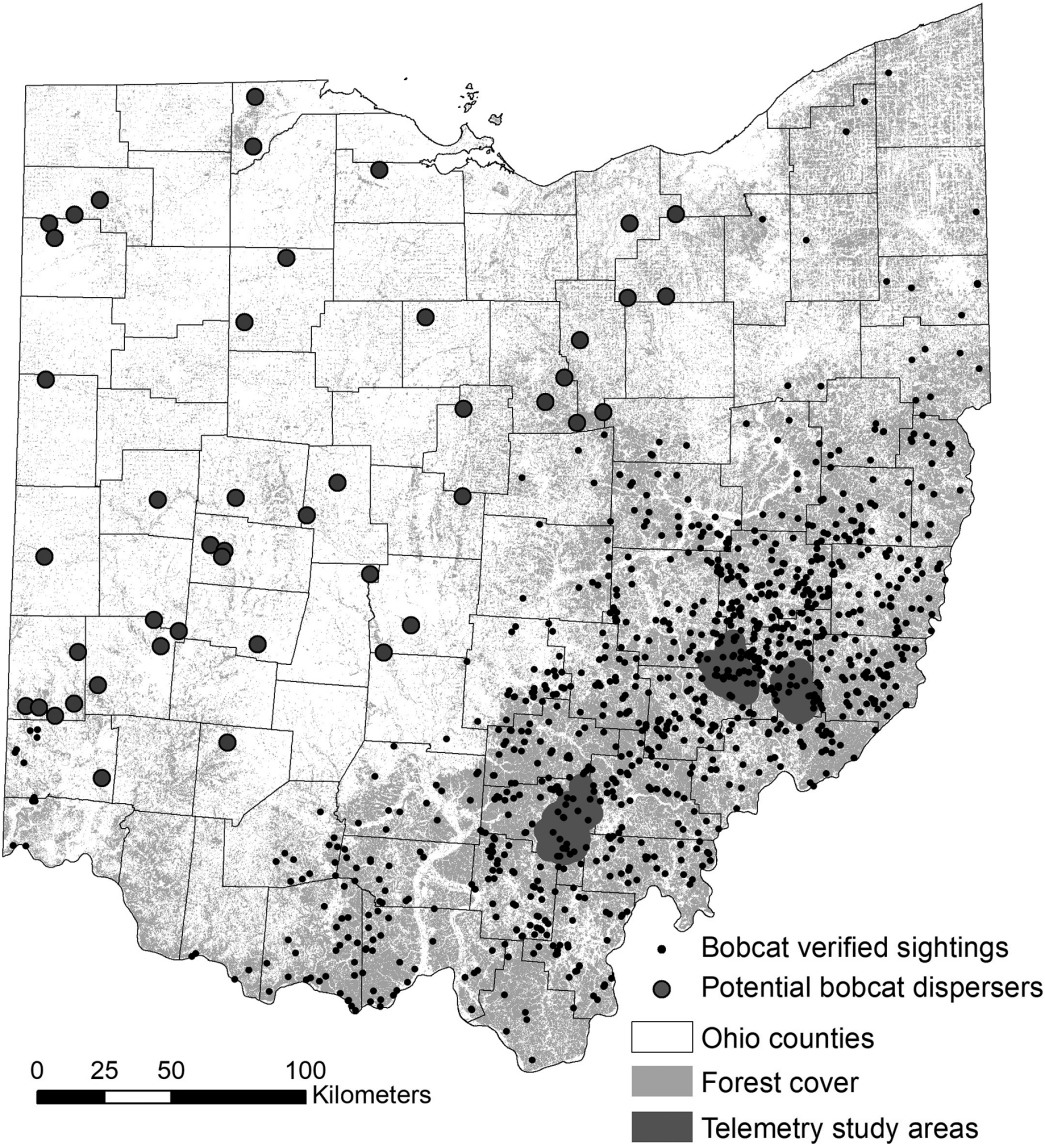
Study area map for bobcat habitat selection analyses. Verified non-roadkill sightings (*n* = 975) of bobcats in Ohio between 2010 and 2019, and two areas where GPS telemetry was implemented between 2012 and 2014. The telemetry areas correspond to genetically distinct eastern and southern sub-populations (Anderson, Prange & Gibbs, 2015).

We used bobcat sightings recorded by the ODOW between the years 2010 and 2019 (Fig. 1). The dataset included multiple types of verified records, such as camera trap images, observations of live animals by wildlife experts, roadkill animals and incidentally trapped animals. The majority of the sightings were reported by the general public, who were encouraged to report all bobcat sightings through the yearly regulations booklet and other ODOW announcements *via* phone, email, or an online form (starting in 2017). The original dataset contained >1,500 reported sightings, and we eliminated all roadkill incidents, which would introduce a bias towards roadways, which is the largest source of bobcat mortality in Ohio (Bencin et al., 2019). After eliminating entries where geospatial data were missing, 975 unique observations remained.

### Telemetry data

Telemetry study areas were located in the southern and eastern regions of the state, and were selected based on the presence of two recognized subpopulations ((Anderson, Prange & Gibbs, 2015; Prange & Rose, 2020); Fig. 1). For the individual-level resource selection analysis, we used a telemetry dataset for 28 bobcats collected between 2012 and 2014. Telemetry data collected was previously described in (Bencin et al., 2019) and (Prange & Rose, 2020). Bobcats were fitted with Telemetry Solutions Quantum 4000 (Telemetry Solutions, San Francisco, CA, USA) or Tellus GPS System (Followit, Lindesberg, SWE) collars that were programmed to locate individuals twice daily at 12-h intervals on a system that rotated through a 24-h period. GPS collars frequently located individuals only once daily, and collar performance varied. We removed several animals from analysis because of (1) a small number of fixes (<30) and (2) dispersal behavior. As such, we focused the analysis on 20 resident animals with >30 GPS fixes (maximum number of fixes was 630); 13 individuals (seven females; six males) occurred in the eastern subpopulation and seven individuals (four females; three males) in the southern subpopulation.

### Habitat selection analyses

#### Population level habitat selection

We used logistic regression (Hosmer & Lemeshow, 1989) with verified sightings as occurrences and four times more random locations as pseudo-absences (Barbet-Massin et al., 2012). We created a 5,600 m buffer around all bobcat sighting locations and generated the random points outside this buffer. The buffer size was equal to the radius of a circle with the same area as the mean bobcat home range in Ohio (99.7 km^2^, (Prange & Rose, 2020)), We evaluated three categories of predictors that potentially affected the population-level resource selection in Ohio: land cover type, distance to road, and distance to forest.

Although bobcats are a habitat generalist and can persist in heterogeneous, mixed-use landscapes, including suburban areas (Beattie, 2020), their primary habitat in the eastern US and Ohio is forest (Linde et al., 2012; Reding et al., 2013; Prange & Rose, 2020), which is congruent with our verified sightings dataset. As such, we wanted to investigate the contribution of forest and other land cover categories to population-level selection. We used the 2016 National Land Cover Dataset (30 m resolution; (Yang et al., 2018; Jin et al., 2019)) and extracted the following categories: forest (all forest types combined), crop, pasture, herbaceous/grassland, shrub and developed (all levels of developed lands). We evaluated the proportion of each habitat type at multiple scales using a moving window approach. These moving window sizes range from areas covered during daily movements (7 km^2^; edge of moving window = 2.64 km (Bencin et al., 2019)) to 15, 30 and 50 km^2^, which represent 1/4, 1/3 and 1/2 of mean core home range sizes of individual bobcats in Ohio, respectively (Prange & Rose, 2020).

Roads are known to influence habitat selection at the individual home range level (Poessel et al., 2014; Litvaitis et al., 2015), and we included distance from high traffic roads (interstate, state routes and US routes) as a variable in the model. Ohio has an overall high-density of roads, and other studies showed that bobcat occupancy was not associated with road density (Rich et al., 2018) and bobcats do not avoid low-traffic roads. However, high traffic roads induce high mortality in Ohio’s bobcat population (Bencin et al., 2019), thus we expect a negative relationship between distance to high traffic roads and habitat suitability.

We also calculated the distance to forest because a previous telemetry study in Ohio showed that bobcats tend to use edges between forest and open habitat for foraging (Prange & Rose, 2020), suggesting that these areas may have high suitability. We investigated the correlations between predictor variables and did not use variables with a Pearson *r* > |0.7| (*e.g*. we removed distance to forest and proportion of cropland from our final model set, as both were highly correlated with proportion of forest at all spatial scales). In addition, because the variables are highly correlated across multiple scales (*e.g*. proportion of forest within 7, 15, 30 and 50 km^2^), none of our models contained the same land cover variable at more than one scale. We scaled and centered the variables prior to modeling using the function *scale* in program R 4.0.3 (R Core Team, 2021). We used logistic regression (Hosmer & Lemeshow, 1989) to build a set of models using combinations of land cover variables at different scales (Table 1) and distance to high traffic roads, and an information-theoretic approach based on Akaike’s Information Criterion corrected for small sample size (AICc) to rank the models (Burnham & Anderson, 2002). We built models using the variables proportion of forest, pasture and herbaceous vegetation at each scale, with and without distance to high traffic roads. We also explored several combinations of variables at different scales. In particular, we used forest at the 50 km^2^ scale in combination with pasture and herbaceous vegetation at finer scales, and also tested the importance of forest at finer scales; the reasoning was that forest is the main driver of bobcats occurrence and persistence in the US Midwest (Kamler & Gipson, 2000; Tucker, Clark & Gosselink, 2008; Linde et al., 2012; Reding et al., 2013), thus forest at the broader scale would be more predictive, while open habitat is important at finer scales (Kamler & Gipson, 2000; Prange & Rose, 2020). We predicted population-level habitat selection probabilities using a model-averaging approach for models within 2 AICc units from the top model (using R package ‘*MuMIn*’ (Barton, 2020)), and mapped the resulting habitat suitability map spatially (using R package ‘*raster*’ (Hijmans, 2020)). We assessed model fit using the Area Under the Curve of the Receiver Operating Characteristic (AUC ROC). This metric ranges between 0.5 (complete randomness; model cannot discriminate between presences and pseudo-absences) and 1 (perfect discrimination between presences and pseudo-absences), with values >0.8 denoting good model fit. We also used a k-fold cross-validation technique to predict predictive ability of our models. We trained the model containing our top predictors on a training dataset (95% of the data) and tested its predictive ability on a test dataset (5% of the data), repeating the procedure 1,000 times. We examined the mean accuracy and the distribution of accuracy values across the 1,000 repeats.

**Table 1:** Model selection table for bobcat population-level habitat selection analyses *via* logistic regression.

Model	#params.	Log-likelihood	Delta AICc	AICc weight	AUC ROC
DistMainRoads + Forest50km + Past50km	4	−1,174.296	0.00	0.472	0.958
DistMainRoads + Forest50km + Past50km + Herb30km	5	−1,174.272	1.95	0.178	0.958
DistMainRoads + Forest50km + Past50km + Herb50km	5	−1,174.280	1.97	0.176	0.958
DistMainRoads + Forest50km + Past50km + Herb15km	5	−1,174.295	2.00	0.174	0.958
DistMainRoads + Forest50km + Past30km + Herb30km	5	−1,182.879	19.17	0	0.957
DistMainRoads + Forest30km + Past50km + Herb30km	5	−1,207.052	67.51	0	0.956
DistMainRoads + Forest30km + Past50km + Herb30km	5	−1,207.725	68.86	0	0.956
DistMainRoads + Forest50km	3	−1,258.790	166.99	0	0.952
DistMainRoads + Forest15km + Past15km + Herb15km	5	−1,270.806	195.02	0	0.952
DistMainRoads + Forest15km + Past30km + Herb30km	5	−1,283.174	219.76	0	0.952
DistMainRoads + Forest7km + Past7km + Herb7km	5	−1,350.636	354.68	0	0.947
DistMainRoads + Forest15km	3	−1,378.350	406.11	0	0.944
DistMainRoads + Past30km + Herb30km	4	−2,543.026	2,737.46	0	0.761
DistMainRoads + Past30km	3	−2,670.971	2,991.35	0	0.737
DistMainRoads	2	−2,859.052	3,365.51	0	0.517
Null	1	−2,867.632	3,380.67	0	0.5

**Note:**

The top four models were used to predict model-averaged habitat suitability for Ohio’s bobcats. AUC ROC is the area under the curve of the receiver operating characteristic.

#### Individual level habitat selection

We used generalized linear models and the package ‘*ResourceSelection*’ (Lele, Keim & Solymos, 2019) for program R 3.6.3 (R Core Team, 2021) to evaluate habitat selection within home ranges. We tested both RSPF (resource selection probability function) and RSF (resource selection function); RSFs are most commonly used in ‘*used-available*’ designs, and yield predictions that are proportional to the probability of selection (*i.e*. relative probabilities of selection (Johnson et al., 2006)). RSPFs have recently emerged as an alternative for estimating the absolute probability of selection of any given spatial unit *via* a ‘*weighted distribution*’ approach that yields stable parameters (Lele & Keim, 2006; Lele, 2009). Although the advantage of RSPFs is producing actual probabilities of selection, the parameters of the exponential RSF are more useful for evaluating the strength of selection across the parameters in the model *via* a Relative Selection Strength (RSS) approach (Avgar et al., 2017). A comparison of the two approaches based on Consistent AIC (CAIC, (Taper, 2004)) is recommended to identify the type of model that fits the data best (Avgar et al., 2017) instead of selecting a method *a priori*.

We created random points within individual home ranges derived from kernel density estimates (within the 95% isopleth). The number of points was equal to the number of GPS telemetry fixes for each individual. This procedure leads to a distribution of data weighted by the data allocation for each individual. We tested two approaches: an unweighted approach that pooled all presence and availability data across all 20 animals (thus effectively ignoring differences in selection between animals), and one in which data were weighted by the individual animals. We examined preliminary distribution-weighted *vs* unweighted models using CAIC (Avgar et al., 2017); distribution weighted models always had a much lower AIC value; thus, we implemented a distribution-weighted approach thereafter. To evaluate the strength of selection for various variables in the model, we adopted an RSS approach (Avgar et al., 2017); specifically, we evaluated the *average* probability of selection across one of the habitat covariates, while averaging over other habitat covariates according to their availability. For this, we implemented the following steps recommended by (Avgar et al., 2017): (1) fit RSF or RSPF; (2) calculate fitted probabilities at *available* locations; (3) plot fitted probabilities for *available* locations against the variable of interest; and (4) fit a smooth nonparametric regression function through these points (done using function *geom_smooth* in package ‘*ggplot2*’ (Wickham, 2016)). This graphical tool showcases the change in the probability of *use* and not the change in the probability of *selection*, thus, it remains robust to new environmental conditions; namely, if the availability of other resources changes, the graph depicting the probability of use also changes.

### Permits/ethics statement

The program administrator for Ohio’s Division of Wildlife, Wildlife Management and Research Group approved the telemetry study along with the agency’s executive administrator for wildlife research, and the wildlife federal aid coordinator (state approval codes: WFSR12 and WFPR18). Bobcat capture and handling techniques were carried out in accordance with the American Society of Mammalogists guidelines. Personnel were trained and supported by a professional USDA APHIS (Animal and Plant Health Inspection Service) trapper, and were Safe-Capture International (Snohomish, WA, USA) certified.

### Connectivity modeling

We investigated the habitat connectivity for bobcats using circuit theory-based software Circuitscape 4.0.5 (McRae et al., 2008; McRae, Shah & Mohapatra, 2013). The strength of a circuit theory approach to modeling connectivity is the ability to simultaneously evaluate contributions of multiple dispersal pathways, as opposed to simpler least-cost path metrics, which yield single connectivity pathways (McRae, 2006; McRae et al., 2008). In Circuitscape, landscapes are represented as conductive surfaces; high conductance/low resistance values are assigned to landscape features that are most permeable to movement or best promote gene flow, and high resistance/low conductance is assigned to barriers to movements.

For the purpose of this study, we decided to adopt a simple approach and used the habitat suitability map resulting from the population-level habitat selection analysis as the input resistance layer. Previous research has shown that habitat suitability can be a good surrogate for explaining functional connectivity and gene flow across landscapes (Wang et al., 2008; Chan, Brown & Yoder, 2011; Gherghel & Martin, 2020). Connectivity modeling using Circuitscape requires nodes, either points or regions between which connectivity is to be modeled. For this analysis, we selected five regions as nodes representing areas that bobcats could disperse to and from in Ohio. These areas were selected at the county level based on whether they represented (1) core areas for genetically distinct eastern and southern subpopulations (Anderson, Prange & Gibbs, 2015): one county in southern Ohio and four counties in eastern Ohio; and (2) counties with recent verified sightings in southwestern (one county), northwestern (one county) and northeastern (one county) Ohio. For this analysis, we were primarily interested in exploring two questions. First, the two genetically distinct subpopulations in the telemetry study were separated by ~200 km, and the area between the subpopulations is predominantly forested with no natural or significant man-made barriers to dispersal. As such, we wanted to evaluate the potential for admixture between the two subpopulations (a separate genetics study of 120 roadkill animals collected throughout southeastern Ohio in 2019–2020 is underway). Second, outside southeastern and southern Ohio, land cover is dominated by agriculture and urban and suburban development (Fig. 1), yet bobcats have been sighted in many areas of the state, indicating dispersal through presumably suboptimal habitat. As such, we wanted to evaluate corridors for dispersal through suboptimal habitat, which could further inform management of the population (*e.g*. viability of lethal management (*i.e*. trapping or hunting) in areas that serve as conduits for dispersal).

We modeled the relationship between habitat suitability and permeability to movement using three functions: a linear function (suitability values were inversely proportional to the resistance values (*i.e*. cells with highest habitat suitability values had the lowest resistance to movement, and *vice versa*)) and two non-linear functions. The non-linear functions took an exponential decay form using the transformations suggested by Keeley, Beier & Gagnon (2016), with an exponent c = 2 and c = 8 determining the shape of the curve (c = 8 assumes that resistance to movement decreases sharply at low suitability values, while c = 2 provides an intermediate relationship between the other two).

$$R = 100 - 99*\;\displaystyle{{1 - {e^{ - c\ *\ H}}} \over {1 - {e^{ - c}}}}$$

where R = resistance, H = suitability, and factor *c* determines the shape of the curve (c = 2 and c = 8 in our case).

We used the resistance maps under the three scenarios to model connectivity as described above, and visualized the connectivity map using a quantile distribution of map values; this visualization approach performs best at showcasing areas of high and limited connectivity, and highlights corridors with high flow in otherwise low permeability landscapes (McRae et al., 2008). To test which relationship between resistance and habitat suitability performed best and produce a final connectivity map informed by biological data, we identified 46 bobcat locations in our dataset that were outside the high suitability regions of the state that could potentially be locations of dispersing individuals. For each of these locations (1’s), we generated three random points within a 5-km buffer (0’s), which represent available locations in the landscape. For both bobcat and random locations, we extracted the quantile (1–10) associated with the connectivity predictions under the three relationships between resistance and suitability. We used a Generalized Linear Mixed Effect Model (GLMM) framework and tested three models; each model had the quantile value for that perspective resistance-suitability relationship as a *fixed effect*, and location ID as a *random effect* (to account for variation within each location). The goal of this procedure was to evaluate which connectivity map was a better predictor for occurrences of potential bobcat dispersers. We ranked the three models using AICc, and extracted the AICc weight for each model in the model set. Lastly, we used the AICc weights to calculate a weighted average of the three maps, and produce a model-averaged connectivity map. GLMM’s were implemented *via* package ‘lme4’ (Bates et al., 2015), and model selection procedure *via* package ‘MuMIn’ (Barton, 2020) in program R 4.0 (R Core Team, 2021).

## Results

### Population-level habitat selection

**Figure 2:**
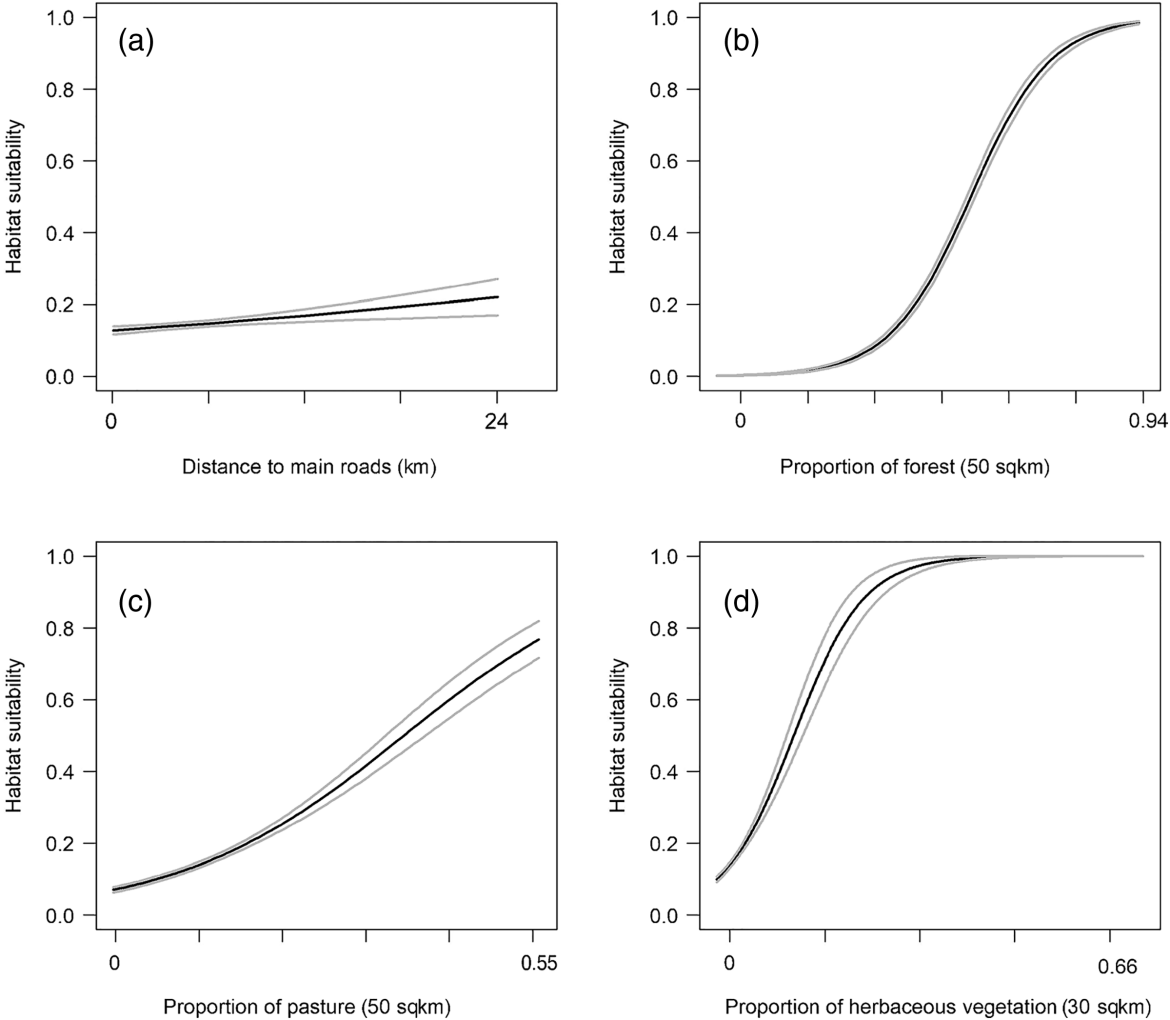
Best predictors for population-level habitat selection by bobcats in Ohio (A–D). We implemented logistic regression using 975 bobcat verified sightings in Ohio (between 2010 and 2019). Black lines are predicted responses to each variable, and gray lines are 95% confidence intervals. The summaries of the four predictors were: proportion of forest within 50 km^2^ = 0–0.94 (median 0.15, mean 0.24, IQR (Interquartile Range) 0.06–0.38); proportion of pasture within 50 km^2^ = 0–0.54 (median 0.07, mean 0.09, IQR 0.02–0.15); proportion of natural herbaceous vegetation within 30 km^2^ = 0–0.64 (median 0.011, mean 0.019, IQR 0.005–0.20); distance to high traffic roads (interstate, state and US routes) = 0–23,373 m (median 3,346, mean 4,272, IQR 1,378–6,257).

**Figure 3:**
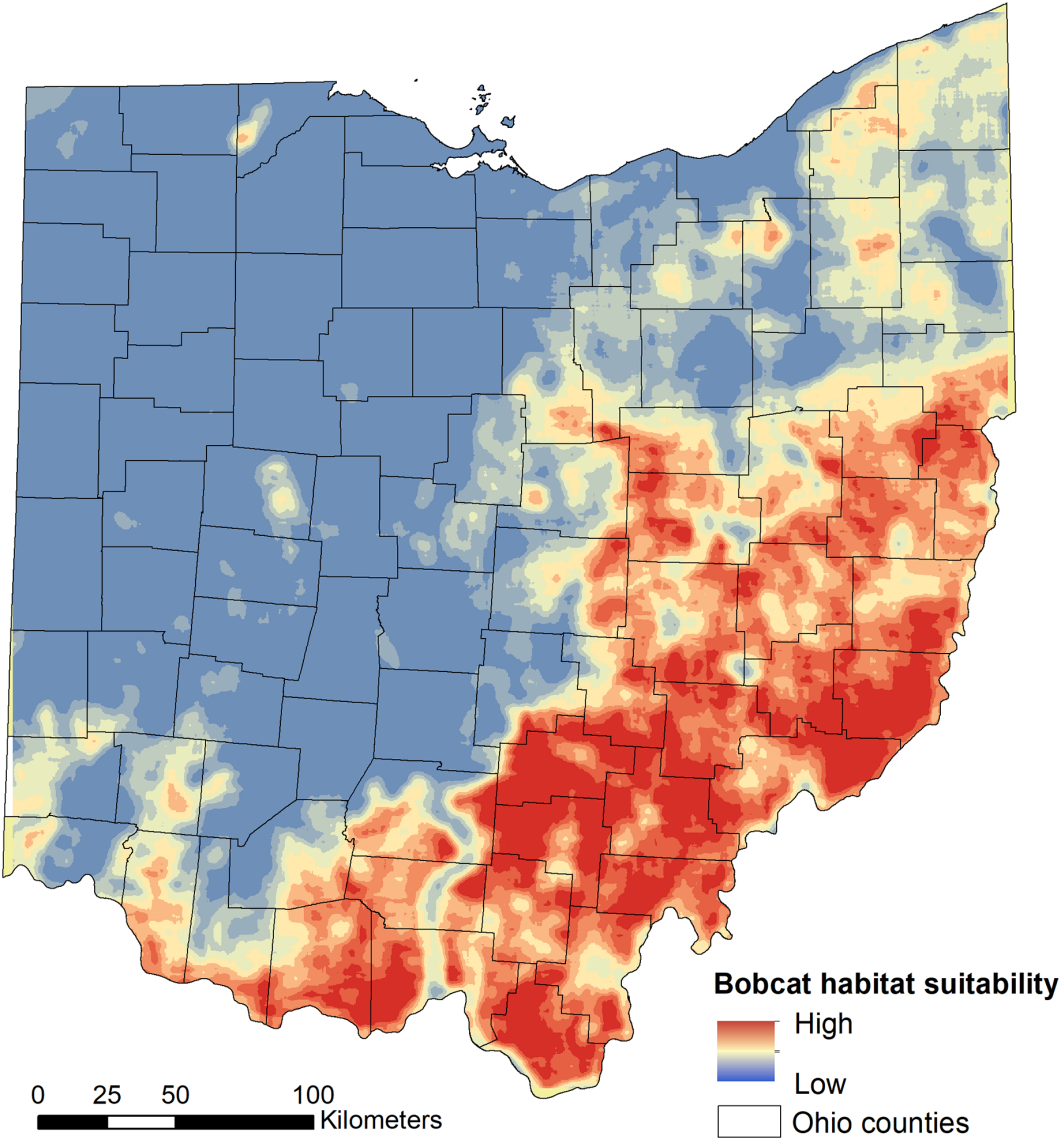
Model-averaged predictions of population-level bobcat habitat selection (suitability). Habitat suitability was modelled using 975 verified sightings collected between 2010 and 2019 using logistic regression.

We found that habitat selection by bobcats in Ohio at the population level was predicted by broad scale habitat variables and distance to main roads (Table 1). The predictive ability of our models was high: average predictive ability based on a k-fold cross validation analysis = 0.94 (Supplemental Material) and AUC ROC values for candidate models used for model averaging was >0.95 (Table 1). Several competing models were within two AICc units from the top model and suggested that the proportion of forest within 50 km^2^ had a strong, positive relationship with habitat suitability (Fig. 2). The proportion of pasture within 50 km^2^ and the distance to high traffic roads also had a positive association with habitat suitability, but not as strong as forest (Fig. 2). The proportion of natural herbaceous vegetation at finer scales (15–50 km^2^) was included in different models within the two AICc model set, in this order, and had a strong, positive association with habitat suitability (Fig. 2; only relationship to natural herbaceous at 30 km^2^ shown). The highest habitat suitability for bobcats was in eastern and southeastern Ohio, which coincides with the areas of forest cover, and bobcat population expansion (Fig. 3). There was high suitability along the southern border with Kentucky and the southwestern Ohio border with Indiana. Central and northwestern Ohio are dominated by cropland, and had low suitability, except for patches of habitat along river corridors; these patches may act as stepping stones for dispersing individuals but are unlikely to be occupied permanently. Northeastern Ohio, which is a mosaic of forest, agriculture and human development, had intermediate suitability (Fig. 3).

### Individual-level habitat selection

The number of bobcat GPS telemetry locations ranged between 31 and 630 locations per individual (Supplemental Material). Females had smaller mean 95% UD home ranges (27.27 km^2^, range = 7.70–77.82 km^2^) compared to males (69.79 km^2^, range = 27.85–165.66 km^2^) (Kruskal–Wallis chi-squared = 6.477, *df* = 1, *p*-value = 0.011). Home ranges were larger in the southern study area (mean = 79.23 km^2^, range = 23.35–165.55 km^2^) compared to the eastern study area (mean = 28.73 km^2^, range = 7.70–8.88 km^2^) (Kruskal–Wallis chi-squared = 5.841, *df* = 1, *p*-value = 0.015).

**Figure 4:**
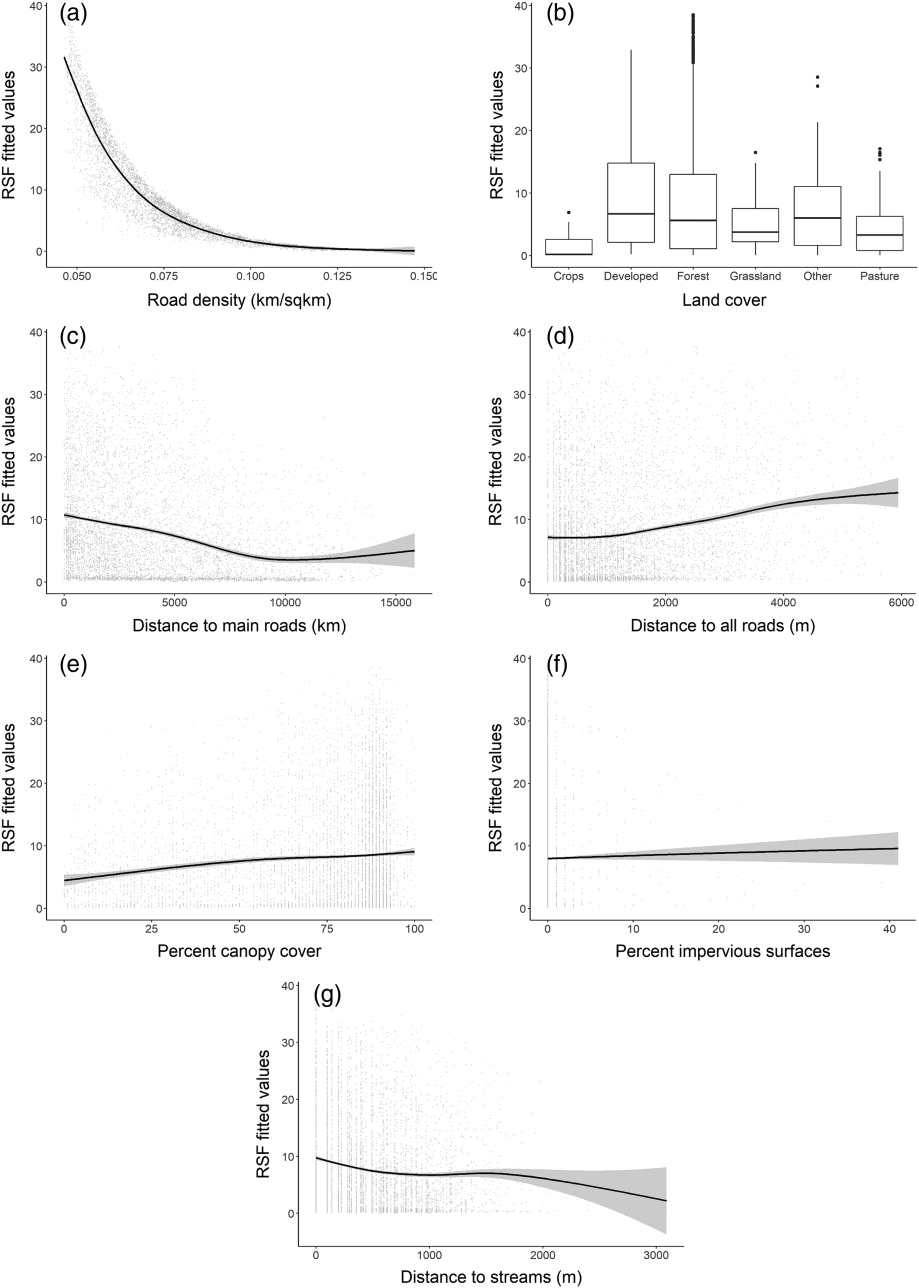
Bobcat habitat selection predictors at the individual (home-range) level (A–G).

The RSF fitted using a weighted-distribution approach with an equal number of locations and random availability points with individual home ranges showed that at the home range scale, bobcats exhibited the strongest selection for areas with low road density (Fig. 4A) and those farther away from main, high-traffic roads (Fig. 4C). Although bobcats also preferred areas with higher canopy cover and a low cover of impervious surfaces, these variables had lower selection strength (for or against; Table 2). Distance to streams and distance to all roads were also weak predictors of selection (Figs. 4D, 4F), relative to road density and high-traffic roads. Land cover played an important role in habitat selection within home ranges, with the strongest selection for forest habitat (Fig. 4B). Bobcats showed weaker selection for open habitat (pasture, grassland, Fig. 4B); although the percent of impervious habitat in a given grid cell was negatively associated with bobcat use, bobcats exhibited selection towards low-intensity developed habitat (Fig. 4B).

**Table 2:** Standardized coefficients of individual-level bobcat habitat selection derived from distribution-weighed exponential RSF models.

Variable	Coefficient (β)	Standard error	*p*-value
Road density	−1.2495	0.0366	<0.0001
% canopy cover	0.0938	0.0202	<0.0001
Distance to main roads	−0.1480	0.0173	<0.0001
Distance to streams	−0.0862	0.0144	<0.0001
Distance to all roads	0.0557	0.0165	0.0007
% impervious surface	−0.0617	0.0147	<0.0001
Land cover (developed)	1.2019	0.1117	<0.0001
Land cover (forest)	1.0617	0.1082	<0.0001
Land cover (grassland)	0.4352	0.1201	0.0003
Land cover (other)	0.7843	0.1308	<0.0001
Land cover (pasture)	0.6522	0.1322	<0.0001

### Habitat connectivity

**Figure 5:**
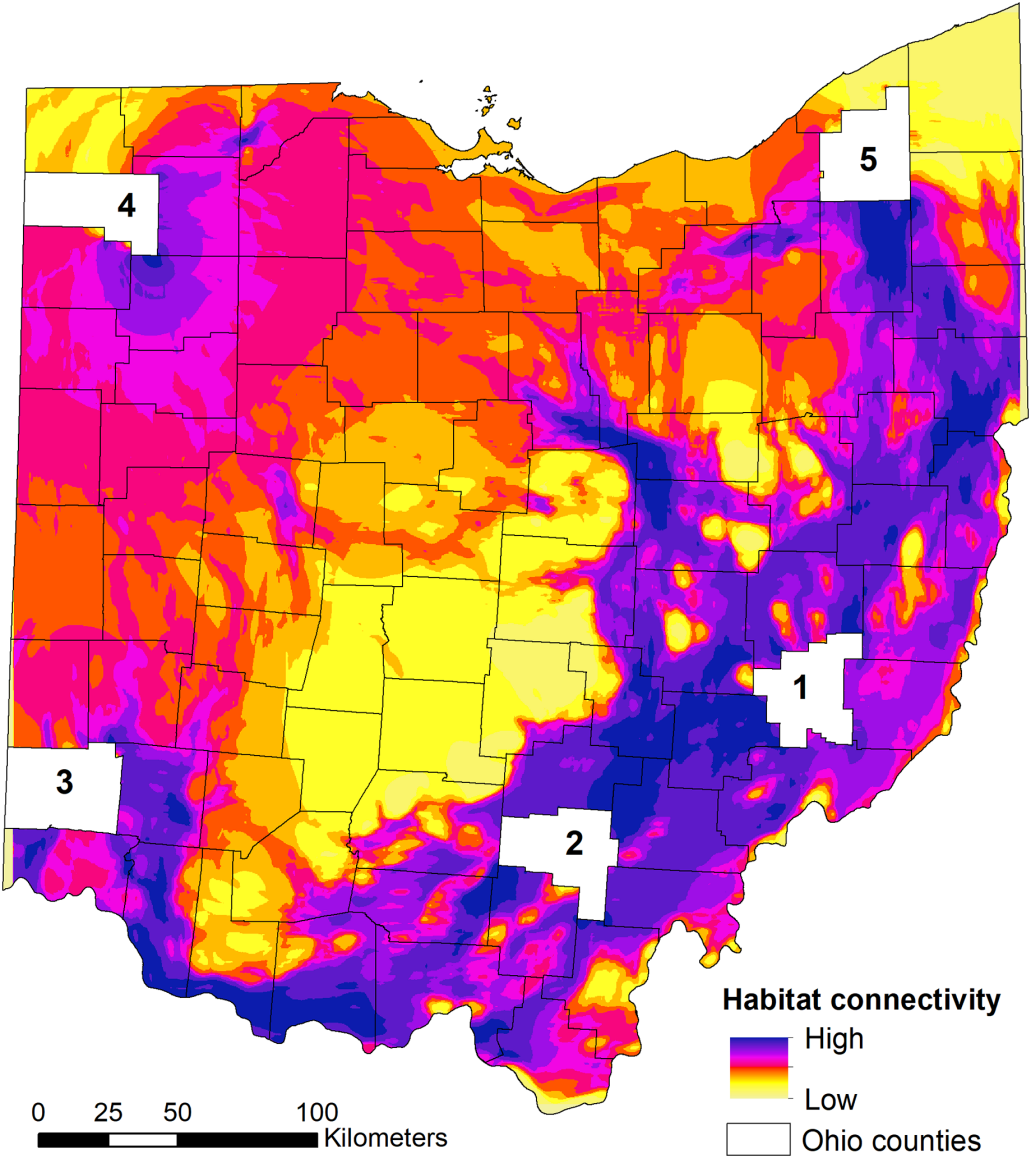
State-wide habitat connectivity for bobcats in Ohio. Connectivity was modeled using Circuitscape and population-level habitat selection outputs (habitat suitability) as the resistance layer (*i.e*. low suitability = high resistance and *vice versa*). The white areas (counties) act as nodes to model potential flow across the landscape using a pairwise approach (between the five counties). The counties were selected based on existing knowledge of genetic differentiation between the eastern (county 1) and southern (county 2) subpopulations; and occurrence of verified sightings in southwestern, northwestern and northeastern areas of Ohio (counties 3, 4 and 5, respectively). Note that low connectivity in the outermost regions (NE, S, NW) are an outcome of the focus on exploring the connectivity between the five regions; this does not mean that these areas completely lack connectivity, but that they just do not contribute to the flow of individuals between the five regions.

We found that the three relationships between habitat suitability and connectivity performed equally well (similar AICc weights) when predicting high connectivity area for dispersing individuals (Table 3). As such, the model-averaged maps were equally weighted in the final connectivity map (Fig. 5). When examining the statewide habitat connectivity for bobcats, several interesting patterns emerged. First, habitat was highly permeable to movements between the core areas of two genetically distinct subpopulations—eastern (region 1) and southern (region 2) (Fig. 5). Connectivity between southern and southwestern Ohio (region 3) was lower and concentrated along the Ohio River Valley; this is likely an outcome of the high proportion of agricultural lands and urban development outside of the river valley (Cincinnati, Dayton and suburbs). Recent sightings in southwestern Ohio could be either animals from neighboring Indiana or Kentucky or dispersers from southeastern Ohio (*e.g*. one GPS-collared subadult male captured in region 2 traveled to the Cincinnati area during the telemetry study). A pattern of high connectivity was evident between eastern and northeastern portions of the state (region 5; Fig. 5). This region is characterized by a mosaic of forest (including the Cuyahoga Valley National Park), agricultural lands, and low-intensity urban development, which allows for many conduits of dispersal. One of the most interesting outcomes of the connectivity analysis was the potential dispersal corridors in central Ohio, to and from the northwestern region (Fig. 5). Within the intensive row-crop agriculture landscape, which is not conducive to bobcat dispersal (Reding et al., 2013), riparian forest cover along water courses could act as conduits for dispersal in this landscape, although overall connectivity was lower compared to the eastern part of Ohio. These areas coincide with several recent verified sightings in central and western Ohio (Fig. 1), and are potential movement corridors across the landscape. Western and northwestern Ohio had relatively low connectivity, spread across a larger area; this area is relatively homogeneous, with agricultural lands interspersed with small forest patches. Lastly, there were several areas with no connectivity in central Ohio; these areas lack forest cover and have intensive agricultural use (Figs. 1, 5).

**Table 3:** Model selection table for logistic regression models predicting habitat connectivity. We used *n* = 46 locations of potential dispersing individuals and random locations (3 per occurrence) within a 5 km circular neighborhood around occurrence points. (lin = linear relation between suitability and resistance; 2 and 8 are *c* exponents for non-linear relationships between suitability and resistance based on Keeley, Beier & Gagnon (2016)). AICc for top model = 212.9.

Model	df	LogLik	Delta AICc	AICc weight
Non-linear relation (c = 8)	3	−103.454	0.00	0.335
Linear	3	−103.458	0.01	0.334
Non-linear relation (c = 2)	3	−103.465	0.02	0.331

## Discussions

Our research represents the first multi-scale assessment of habitat selection and connectivity for bobcats in a recently recolonized landscape in the Midwestern United States. Using multiple data sources, including citizen science observations and telemetry data, we found that bobcats in Ohio are currently largely limited to the forested part of the state, and that proportion of various land cover types (forest, herbaceous, and pasture) at broad spatial scales (30 km^2^ and 50 km^2^) had a positive association with statewide bobcat selection and habitat suitability. Bobcat observations outside the high suitability area have been recorded in the last several years (since 2016–2017) suggesting that animals are dispersing across Ohio in areas of low predicted suitability. At the finer, individual-level selection, GPS telemetry data showed that, within home ranges, bobcats selected for areas with low road density and away from high traffic roads, while still largely selecting for forested habitat. Statewide connectivity, modeled as a weighted average of predictions based on three types of relationships between habitat suitability and resistance to movements, showed that connectivity was highest in the eastern and southern parts of Ohio, while central, western and northwestern Ohio has lower connectivity. Bobcat occurrences have been recorded in medium connectivity areas, sometimes related to riparian corridors in mixed agricultural landscapes, suggesting that areas with low suitability are important for statewide population dynamics *via* dispersal processes.

### Population-level selection

Unsurprisingly, the highest probability of bobcat occurrence (*i.e*. suitability) was in the forested southeastern part of Ohio. Eastern and southern Ohio regions are home to two genetically distinct (as of 2012) sub-populations (Anderson, Prange & Gibbs, 2015), and the current population is thought to have expanded from these two regions (roughly, areas 1 and 2 in Fig. 5); preliminary data suggest that as of 2020, the two subpopulations are no longer distinct (V Popescu, 2021, unpublished data). The earliest modern-day records of bobcats in Ohio occurred in these regions, which are in close proximity to well-established populations in Kentucky and West Virginia, and they have continued to have the most consistent sightings over the past several decades (Fig. 1). Ohio bobcats selected for areas with highest forest cover (Fig. 2A), which corroborates other bobcat populations in Midwestern US states with similar history of recovery (*e.g*. Illinois and Iowa; (Nielsen & Woolf, 2002b; Woolf et al., 2002; Reding et al., 2013)). These findings are very different than heavily forested portions of their range (*e.g*. New England), where bobcat habitat suitability was largely driven by snowpack depth (Reed et al., 2017b). However, we also found that bobcats selected for higher proportions of natural herbaceous vegetation, as well as pasture (Figs. 2B, 2C). As a species with high flexibility in habitat selection at the population scale (bobcats have a large distribution in North America overlapping a broad range of habitat types; (Roberts & Crimmins, 2010; Dyck, Wyza & Popescu, 2021)), bobcats can take advantage of existing habitat resources and be successful in a variety of natural, rural and suburban settings (Kamler & Gipson, 2000; Linde et al., 2012; Beattie, 2020).

Our models predicted that northern and northeastern Ohio had low-medium suitability. While our dataset did not include many bobcat occurrences in these regions, bobcats are known to thrive in heterogeneous, human-dominated landscapes, and recent observations of animals in these areas are increasing. The increasing number of observations in peripheral areas is concomitant with an increase in observations in SE Ohio, and could also be an artifact of greater availability and affordability of monitoring technology (*i.e*. trail cameras), which also likely led to increased reporting of animals. Bobcats in other US Midwest regions (Tucker, Clark & Gosselink, 2008; Reding et al., 2013) and the Eastern US (Beattie, 2020) were found to be resident of heterogeneous mixed-use and suburban landscapes, which may include lower suitability areas. Given that the population has likely not yet reached equilibrium and continues to expand, it is likely that bobcats can take advantage of resources in this region composed of a mosaic of forest, agriculture and suburban development. Moreover, bobcat densities in heterogeneous landscapes (including suburban and forest interspersed with open natural habitat) may reach higher levels compared to forested landscapes; early seral habitats likely have higher prey availability compared to mature forest, which results in smaller home ranges (Tucker, Clark & Gosselink, 2008; Prange & Rose, 2020) and potentially higher density. Because bobcat occupancy and density are linearly and positively related (Clare, Anderson & MacFarland, 2015), it is important to continue to collect bobcat occurrence data and update the population-level selection models periodically to reflect future expansion in areas currently predicted as low-medium suitability.

Habitat suitability in the central, western and northwestern part of Ohio was low due to the landscape characterized by intensive agriculture with little natural habitat remaining. Bobcat observations in these areas were rare, only occurred in the last 3–4 years of the dataset and likely reflect dispersal events. These observations are important from two perspectives; first, they showcase the ongoing expansion process, which makes the case for monitoring these areas closely to evaluate residency (*e.g*. recording females with kittens) and for updating the population-level selection in the future; second, there is a need to understand the selection of dispersal habitat and if, or how, it differs from selection of residential habitat (Reding et al., 2013). The Ohio bobcat population is part of the broader US Midwestern and Eastern population; while genetic differences exist between populations at a regional level (Anderson, Prange & Gibbs, 2015), long-distance dispersal movements do occur, even in unsuitable landscapes that pose high resistance to movement. Bobcats may make use of forested riparian corridors or forest patches embedded in agricultural landscapes, thus maintaining the integrity of such features could be critical for the regional population.

### Individual-level selection

Habitat selection at the individual animal level based on GPS telemetry of 20 adults (11 females and nine males) in two areas of Ohio that coincided with genetically distinct populations revealed complex responses to various cover types, topography, and human disturbance. An earlier study of habitat selection using a slightly larger version of the same dataset used here and Manly’s selection ratios (Prange & Rose, 2020) showed that adult bobcats selected for forest habitat in the southern population and heterogeneous landscapes resulted from reclaimed coal strip-mining in the eastern population (Fig. 1). Reclaimed strip mine lands provide a mosaic of forest and natural herbaceous vegetation that may provide more foraging opportunities and prey items compared to closed-canopy forest habitat (Rose & Prange, 2015; Prange & Rose, 2020). Our findings corroborate current studies, and also adds other evidence for home range scale selection, particularly related to artificial features. Bobcats in our study area selected for habitats with less road density, and farther away from high traffic roads (interstate, state and US routes), and showed a positive association with lower-traffic roads (including unpaved roads). These patterns of habitat use in relation to roads have been found in other telemetry studies across the bobcat range in North America (New Hampshire (Broman et al., 2014; Reed et al., 2017a); California (Poessel et al., 2014)). The information provided by the individual-level selection complements the population-level selection findings and has value when considering habitat connectivity, as discussed below. High traffic roads were a negative predictor at both population and individual-level selection analyses. While bobcats persist in human-dominated landscapes, including suburban, these findings, along with avoidance of high road density areas, suggest that areas away from high-traffic roads and with low road density are important for population persistence. High traffic roads not only affect habitat suitability statewide, but also bobcat activity at home range level, and have negative effects on population demography *via* road mortality (Litvaitis et al., 2015; Bencin et al., 2019).

### Habitat connectivity

Our assessments of habitat connectivity corroborate those of Reding et al. (2013), who studied bobcat habitat resistance to movement using genetic data. They found that in forested areas, the landscape was permeable to movement, movements were determined by landscape composition (*i.e*. availability of forest), and that Euclidean distance did not explain genetic connectivity. For example, we found high connectivity between the eastern population core (region 1 in Fig. 5) and northeastern Ohio (region 5), suggesting that the paucity of observations in this area is not due to a lack of dispersal potential, but likely stems from lower overall suitability and the fact that the population is yet to reach equilibrium and achieve its full distribution in Ohio. We also arrived to similar conclusions concerning low connectivity in the intensive, row-crop agriculture and urban landscape of central and western Ohio. These areas have both low habitat suitability, and low permeability to bobcat movements (relative to the forested areas; Hughes et al., 2019), as well as urban centers, which have been shown to act as barriers to movement in some cases (Reed et al., 2017a). For example, Hughes et al. (2019) found that intensive row-crop agriculture regions of Iowa pose a barrier to movement of dispersing animals, thus limiting the expansion of the Iowa bobcat population.

Although Ohio is one of the most road-dense states in the United States, and roads can be a significant source of mortality for bobcats (Litvaitis et al., 2015; Bencin et al., 2019), we did not include roads as potential barriers in the connectivity models. Bobcat dispersal movements are typically performed by 1- and 2-year old individuals (Nielsen & Woolf, 2003; Johnson, Walker & Hudson, 2010), and are biased towards males (in Iowa, 65% of males and 26% of females dispersed; Hughes et al., 2019). These studies found that bobcats can travel hundreds of kilometers through a variety of habitats, and roads, including high traffic interstate routes, were not a barrier or pose resistance to bobcat dispersal movements (Nielsen & Woolf, 2003). However, dispersing bobcats, particularly males, are susceptible to higher road mortality; an ongoing analysis of 300 roadkill individuals in Ohio between 2010 and 2020 showed that 60% of animals were males and roadkill proportion was twice the stable stage distribution for 1-year old animals (M Dyck, 2021, unpublished data).

The habitat resistance-suitability relationships are still the subject of ongoing research. As such, imposing resistance-to-movement values in environmental layers is highly controversial, and different approaches may yield vastly different results (see Reed et al. (2017b) for an example on habitat connectivity modeling for bobcats in New Hampshire using different data sources). Optimization techniques based on genetic information can be used to determine the best environmental layers for modeling connectivity (Peterman, 2018). However, habitat models have been shown to perform well in explaining dispersal and gene flow across landscapes (Wang et al., 2008), and in the absence of information on habitat use by dispersers.

## Conclusions

In summary, melding the two habitat selection analyses with connectivity modeling also revealed the potential for bobcat population expansion. The population-level selection model reflects the current status of population expansion, the individual-level selection showcases habitat pre-requisites for bobcat persistence at a local level, while the connectivity model sheds light on pathways for future expansion across the state. The current information is aimed at supporting immediate management decisions for bobcats in our study region, and also providing evidence for regional population dynamics in Ohio and surrounding states. We recommend that the suitability and connectivity models should be periodically updated until the population reaches an equilibrium, and also expanded to a broader spatial extent that includes both states with recovering populations and states that served as sources of colonists for the US Midwest bobcat population.

## Supplemental Information

10.7717/peerj.12460/supp-1 Supplemental Information 1 Ohio bobcat verified sightings data 2010–2019. Bobcat verified sightings from citizens used to evaluate population-level habitat selection.

10.7717/peerj.12460/supp-2 Supplemental Information 2 Number of GPS locations and 95% Kernel Density Estimate home ranges for 20 male and female bobcats. The 95% home range boundaries were used in the individual-level resource selection analysis to estimate the availability of environmental gradients and habitat types.

10.7717/peerj.12460/supp-3 Supplemental Information 3 Ohio bobcat GPS telemetry dataset.

10.7717/peerj.12460/supp-4 Supplemental Information 4 R code and results for k-fold cross validation for predicting model accuracy.

## Additional Information and Declarations

### Data Availability

The following information was supplied regarding data availability: The raw data is available in Supplemental Files.
