# Supplementary material for: Habitat connectivity and resource selection in an expanding bobcat (Lynx rufus) population
Source: PeerJ. 2021 Nov 11;9:e12460. doi: 10.7717/peerj.12460 (PMC8590802; doi:10.7717/peerj.12460)
Supplement: Supplemental Information 2 — The 95% home range boundaries were used in the individual-level resource selection analysis to estimate the availability of environmental gradients and habitat types. [file peerj-09-12460-s002.docx]

Number of GPS locations and 95% Kernel Density Estimate home ranges for 20 male and female bobcats. The 95% home range boundaries were used in the third-order resource selection analysis to estimate the availability of environmental gradients and habitat types.

| **Bobcat ID** | **Sex** | **Region** | **95% UD (km^2^)** | **Number GPS locations** |
| --- | --- | --- | --- | --- |
| 11 | M | E | 31.076 | 147 |
| 15 | F | E | 11.601 | 630 |
| 16 | F | E | 7.702 | 354 |
| 19 | M | E | 28.145 | 301 |
| 20 | F | E | 15.189 | 340 |
| 21 | M | E | 27.857 | 273 |
| 22 | M | E | 88.880 | 189 |
| 23 | F | E | 23.450 | 138 |
| 24 | F | E | 11.849 | 45 |
| 2 | F | E | 14.118 | 34 |
| 3 | M | E | 48.227 | 31 |
| 6 | M | E | 54.182 | 370 |
| 8 | F | E | 11.204 | 449 |
| 4 | F | S | 36.809 | 369 |
| 5 | M | S | 165.552 | 346 |
| 10 | F | S | 66.865 | 369 |
| 12 | F | S | 77.817 | 558 |
| 14 | F | S | 23.355 | 559 |
| 17 | M | S | 152.571 | 292 |
| 18 | M | S | 31.619 | 406 |
