# Supplementary material for: Habitat connectivity and resource selection in an expanding bobcat (Lynx rufus) population
Source: PeerJ. 2021 Nov 11;9:e12460. doi: 10.7717/peerj.12460 (PMC8590802; doi:10.7717/peerj.12460)

**R code and results of k-fold cross validation for predicting model accuracy**

Pseudo-R code adapted from <https://www.r-bloggers.com/2015/09/predicting-creditability-using-logistic-regression-in-r-cross-validating-the-classifier-part-2-2/>

# extract dataset with presence/absence and variables used to build a model

dat <- subset(…)

str(dat)

# False positive rate

fpr <- NULL

# False negative rate

fnr <- NULL

# Number of iterations

k <- 1000

# Initialize progress bar

pbar <- create_progress_bar('text')

pbar$init(k)

# Accuracy

acc <- NULL

for(i in 1:k) {

# Train-test splitting

# 95% of samples -> fitting

# 5% of samples -> testing

smp_size <- floor(0.95 * nrow(dat))

index <- sample(seq_len(nrow(dat)),size=smp_size)

train <- dat[index, ]

test <- dat[-index, ]

# Fitting our model with best predictors

model <- glm(Presence ~ forest_50ks + pasture_50ks + herbscrub_30ks + distrds,

family=binomial,data=dat)

# Predict results

results_prob <- predict(model,subset(test,select=c(2:5)),type='response')

# If prob > 0.5 then 1, else 0

results <- ifelse(results_prob > 0.5,1,0)

#str(results)

results <- as.factor(results)

# Actual answers

answers <- test$Presence

#str(answers)

answers <- as.factor(answers)

# Accuracy calculation

misClasificError <- mean(answers != results)

# Collecting results

acc[i] <- 1-misClasificError

# Confusion matrix

cm <- confusionMatrix(data=results, reference=answers)

fpr[i] <- cm$table[2]/(nrow(dat)-smp_size)

fnr[i] <- cm$table[3]/(nrow(dat)-smp_size)

pbar$step()

}

# Average accuracy of the model

**mean(acc) # 0.944**

par(mfcol=c(1,2))

# Histogram of accuracy

hist(acc,xlab='Accuracy',ylab='Freq',

col='cyan',border='blue',density=30)

# Boxplot of accuracy

boxplot(acc,col='cyan',border='blue',horizontal=T,xlab='Accuracy',

main='Accuracy CV')

# Confusion matrix and plots of fpr and fnr

mean(fpr)

mean(fnr)

hist(fpr,xlab='% of fpr',ylab='Freq',main='FPR',

col='cyan',border='blue',density=30)

hist(fnr,xlab='% of fnr',ylab='Freq',main='FNR',

col='cyan',border='blue',density=30)


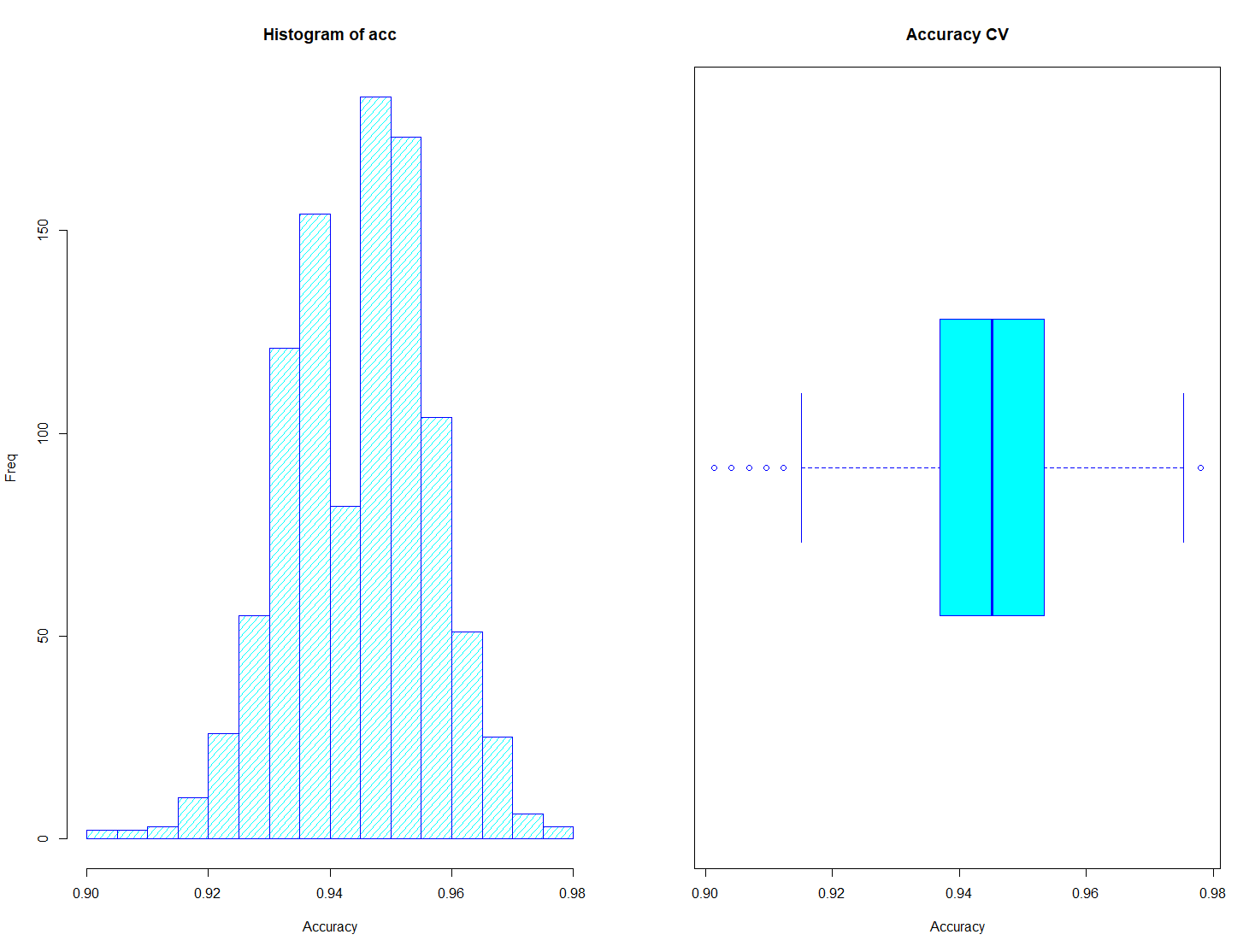

Supplement: Supplemental Information 4 [file peerj-09-12460-s004.docx]
